# Fundamental Understanding and Research Progress on the Interfacial Behaviors for Potassium‐Ion Battery Anode

**DOI:** 10.1002/advs.202200683

**Published:** 2022-05-09

**Authors:** Fei Yuan, Zhaojin Li, Di Zhang, Qiujun Wang, Huan Wang, Huilan Sun, Qiyao Yu, Wei Wang, Bo Wang

**Affiliations:** ^1^ Hebei Key Laboratory of Flexible Functional Materials School of Materials Science and Engineering Hebei University of Science and Technology Shijiazhuang 050000 China; ^2^ State Key Laboratory of Explosion Science and Technology School of Mechatronical Engineering Beijing Institute of Technology Beijing 100081 China; ^3^ School of Metallurgical and Ecological Engineering University of Science and Technology Beijing Beijing 100083 China

**Keywords:** anode, diffusion kinetics, potassium‐ion batteries, rate capability, two phase interfaces

## Abstract

Potassium‐ion batteries (PIBs) exhibit a considerable application prospect for energy storage systems due to their low cost, high operating voltage, and superior ionic conductivity. As a vital configuration in PIBs, the two‐phase interface, which refers to K‐ion diffusion from the electrolyte to the electrode surface (solid–liquid interface) and K‐ion migration between different particles (solid–solid interface), deeply determines the diffusion/reaction kinetics and structural stability, thus significantly affecting the rate performance and cyclability. However, researches on two‐phase interface are still in its infancy and need further attentions. This review first starts from the fundamental understanding of solid–liquid and solid–solid interfaces to in‐depth analyzing the effect mechanism of different improvement strategies on them, such as optimization of electrolyte and binders, heterostructure design, modulation of interlayer spacing, etc. Afterward, the research progress of these improvement strategies is summarized comprehensively. Finally, the major challenges are proposed, and the corresponding solving strategies are presented. This review is expected to give an insight into the importance of two‐phase interface on diffusion/reaction kinetics, and provides a guidance for developing other advanced anodes in PIBs.

## Graphical abstract

This review first starts from the fundamental understanding of solid–liquid and solid–solid interfaces to in‐depth analyzing the effect mechanism of different improvement strategies on them, such as optimization of electrolyte and binders, heterostructure design, modulation of interlayer spacing, etc. Afterward, the major challenges are proposed, and the corresponding solving strategies are presented.

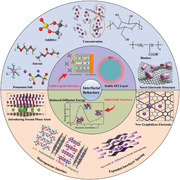

## 1 Introduction

Lithium‐ion batteries (LIBs) have dominated the energy market since they are commercialized;^[^ ^1^, ^2^, ^3^, ^4^ ^]^ however, high cost and uneven distribution of lithium resources greatly hinder the future development of LIBs.^[^ ^5^, ^6^, ^7^ ^]^ Thus, exploring new energy storage technologies based on low cost and high performance is indispensable and urgent. Among them, potassium‐ion batteries (PIBs) are a promising alternative to the start‐of‐the‐art LIBs, due to their unique advantages of abundant potassium resources (2.09 wt%),^[^ ^8^, ^9^, ^10^, ^11^ ^]^ similar “rocking chair” working mechanisms to LIBs,^[^ ^12^, ^13^, ^14^ ^]^ and smaller Stoke's radius in the electrolyte.^[^ ^12^, ^15^, ^16^ ^]^ Besides, the lower standard electrode potential of the K/K^+^ electrode in carbonate ester electrolyte solution is proven before than that of Li/Li^+^, which leads to lower cutoff potentials of the available anode electrodes without metallic potassium deposition, thus PIBs have a potential to realize a higher‐voltage operation in the wider voltage range.^[^ ^17^, ^18^, ^19^ ^]^ Note that the low‐cost and lightweight aluminum foil instead of copper foil can be directly used as an anode current collector in PIBs, contributing to increasing gravimetric energy density and reducing the fabrication cost of PIBs.^[^ ^15^, ^20^ ^]^ Accordingly, in order to make full use of these merits toward high‐performance PIBs, scientific and engineering strategy design is highly demanded.

In recent years, there is a rapid number increase of publications involving PIBs, especially the research on PIB anode, and the resulting PIBs have obvious improved electrochemical performance. Therefore, PIBs are expected to store intermittent electricity for smart grid regulation, and be used as a power source for continuous electricity supply.^[^ ^21^, ^22^, ^23^ ^]^ As we all know, the interfacial behaviors, namely K‐ion diffusion from the electrolyte to the electrode surface (solid/liquid interface), and K‐ion migration between different active particles (solid/solid interface),^[^ ^24^, ^25^, ^26^, ^27^ ^]^ usually play an essential role in thermodynamic and diffusion/reaction kinetics. An improved interface property is expected to greatly boost ion diffusion and electron transport ability as well as maintain structure stability, thus contributing to promoting rate, cycling performance, and Coulombic efficiency (CE). The performances or behaviors of solid/liquid interface are usually related to the stability of solid electrolyte interface phase (SEI) layer, but large volume variations, particle pulverization, and maldistribution of electrode materials may cause the instability of SEI film.^[^ ^28^, ^29^ ^]^ While solid/solid interface properties are often affected by the high transport resistance and low electrochemical reaction.^[^ ^15^, ^30^ ^]^ Consequently, exploring interfacial transmission mechanisms and controlling interfacial transport to improve diffusion/reaction kinetics are highly desirable, which are highly responsible for enhanced electrochemical performance in terms of cycling stability and rate capability.

**Figure 1:**
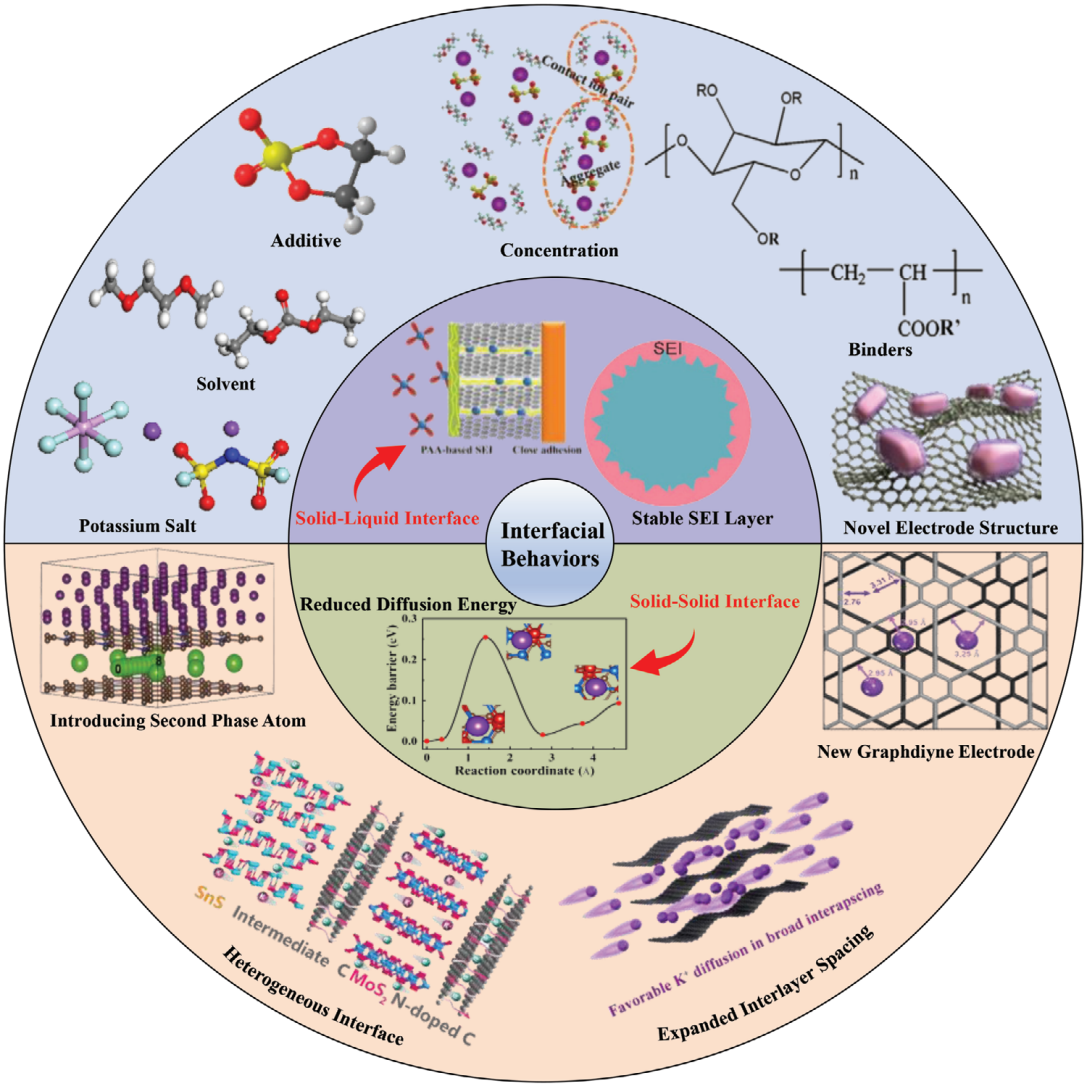
Schematic diagram of the available potassium salt, solvent, additive, electrolyte concentration, binders, electrode structure, etc., for PIBs summarized from the recent literatures.

Considering the practical application, in this review, we start from the fundamental understanding on solid–liquid interface and solid–solid interfaces to discuss the strategies that improve their diffusion/reaction kinetics, mainly including constructing a stable SEI layer and enhancing the diffusion ability of K ions within electrode. Moreover, based on the factors that affect SEI layer (potassium salt species, solvent species, electrolyte concentration/additive species, binder species, etc.), and diffusion capability in electrode (heterostructure, expanded interlayer spacing, etc.) (**Figure** **1**), we also briefly analyze the research progress of interfacial effect on electrochemical performance in PIBs. In addition, some challenges in the optimization of interfacial behaviors are given. This work may help to offer a guidance for understanding the phenomenon and laws that improve electrochemical properties of electrode materials by optimizing interface interaction.

## 2 Research on Solid/Liquid Interface

As we all know, solid/liquid interface usually refers to the electrode/electrolyte interface, namely SEI layer that is derived from the reaction between the anode and the electrolyte. The formed SEI layer can allow the diffusion of metallic cations to maintain the reaction kinetics, and simultaneously insulate the electronic contact between electrode and electrolyte.^[^ ^31^, ^32^ ^]^ It is well accepted that SEI layer plays a vital role in determining initial capacity loss, self‐discharge behavior, cycle, and rate performance. And the composition, structure, and thickness of SEI determine the ability of ion transport. Especially, a stable SEI film formed in a suitable electrolyte can avoid the occurrence of side reaction, while unstable SEI layer inevitably causes the continuous consumption of electrolyte, thus resulting in poor interfacial behaviors.^[^ ^13^, ^15^, ^33^ ^]^ Generally, the key factors that affect SEI are mainly potassium salts, solvents, concentration, additives, binders, and electrode design. Therefore, constructing a stable, dense, and even SEI film is of great significance for achieving excellent interface diffusion based on the optimization of above parameters.

### 2.1 Potassium Salt Species

Potassium salt is one of the vital components of electrolyte system, and has a substantial effect on SEI layer. When selecting potassium salt, some points need to be paid attention to. For example, a high solubility of potassium salt in solvents can increase the concentration of K^+^ in the electrolyte, a good chemical stability can reduce the occurrence of side reactions to some extent, and an excellent thermal stability will promote the safety of battery. At present, there are some potassium salts have been demonstrated in PIBs, such as potassium hexafluorophosphate (KPF_6_), potassium bis(fluorosulfonyl) (KFSI), potassium bis(trifluorosulfonyl)imide (KTFSI), potassium perchlorate (KClO_4_), potassium tetrafluoroborate (KBF_4_), and potassium trifluoromethanesulfonate (KCF_3_SO_3_).^[^ ^34^, ^35^ ^]^ In **Table** **1**, the key physicochemical properties of the potassium salts for PIB electrolytes are summarized.^[^ ^36^, ^37^ ^]^ Among them, due to the low solubilities in typical aprotic solvents and disappointing ionic conductivities,^[^ ^37^ ^]^ KBF_4_ and KClO_4_ receive less attention, and the high cost impedes the wide application of KTFSI and KCF_3_SO_3_. Note that high ionic conductivity makes KPF_6_ and KFSI commonly used potassium salts for PIB anodes.

**Table 1:** Physical and chemical properties of the reported potassium salts for PIB electrolytes^[^ ^36^, ^37^ ^]^

Potassium salts	Molar mass [g mol^−1^]	Decomposition temperature [°C]	Conductivity [mS cm^−1^]	Cost	Solubility	Toxicity
KPF_6_	184.06	575	5.75	Low cost	0.9 mol kg^−1^ in PC; 1.8 mol kg^−1^ in DME	Low toxicity
KFSI	219.23	102	7.2	High cost	Hardly dissolved in PC	Nontoxic
KTFSI	319.24	198–203	6.1	High cost	Hardly dissolved in PC	Nontoxic
KClO_4_	138.55	610	1.1	Low cost	10 mol kg^−1^ in PC; 7.5 mol kg^−1^ in DME	Highly toxic
KBF_4_	125.90	530	0.2	Highly cost	6 mol kg^−1^ in DME	Highly toxic
KCF_3_SO_3_	188.17	238.5	–	High cost	22 mol L^−1^ in water	Nontoxic

**Figure 2:**
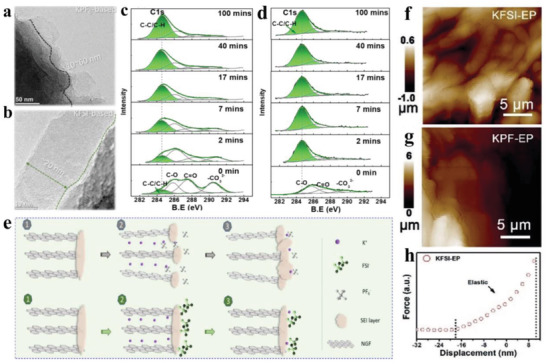
TEM images of NGF electrodes in a) KPF_6_‐based and b) KFSI‐based electrolytes after discharging to 0.01 V (20th discharge). C 1s XPS spectra for c) KPF_6_‐based and d) KFSI‐based electrolytes at different times. e) Schematic illustration of SEI layer formation in KPF_6_‐ and KFSI‐based electrolytes. Reproduced with permission.^[^ ^42^ ^]^ Copyright 2019, American Chemical Society. atomic force microscope (AFM) images of NCS@RGO‐2 electrodes using f) KFSI–EP and g) KPF–EP. h) Representative force–displacement curves of NCS@RGO‐2 electrodes using KFSI–EP electrolytes after 50 cycles. Reproduced with permission.^[^ ^45^ ^]^ Copyright 2018, Elsevier Ltd.

**Figure 3:**
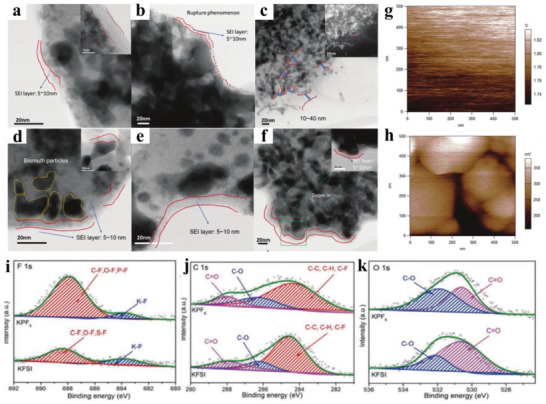
Ex situ HRTEM images of Bi/RRO electrode after two cycles, five cycles, and 10 cycles in a–c) KFP_6_ and d–f) KFSI electrolytes. Surface potential maps of Bi/RGO electrode for g) KPF_6_ electrolyte and h) KFSI electrolyte, respectively. XPS surface analysis for i) F 1s, j) C 1s, and k) O 1s of Bi/RGO electrode after 10 cycles in KPF_6_ and KFSI electrolytes, respectively. Reproduced with permission.^[^ ^46^ ^]^ Copyright 2018, Wiley‐VCH.

Especially, it has been proved by X‐ray photoelectron spectroscopy (XPS) and energy dispersive spectroscopy (EDS) that the KFSI‐induced SEI film is mainly composed of inorganic salt with even distribution features, while KPF_6_ salt often leads to the formation of organic SEI layer.^[^ ^31^, ^38^, ^39^, ^40^ ^]^ In most cases, the SEI layer induced by KFSI is more stable and uniform, and once this SEI membrane is formed, it can inhibit the continuous decomposition of the electrolyte,^[^ ^30^, ^41^ ^]^ resulting in improved interfacial behaviors. Shen and co‐workers^[^ ^42^ ^]^ compared the SEI formation behavior on nitrogen‐doped graphite foams (NGFs) in 0.6 m KPF_6_ in ethylene carbonate (EC)/diethyl carbonate (DEC) (1:1 in volume) and KFSI in EC/DEC (1:1 in volume). As shown in **Figure** **2**a,b, transmission electron microscopy (TEM) images indicate that the SEI layer formed in KPF_6_ has a thickness of 10–60 nm, while the thickness of KFSI‐induced SEI is about 20 nm. Through comparing C—C peak intensity (Figure 2c,d), it is once again revealed that the formed SEI layer in KFSI is more robust and thinner than that in KPF_6_. The schematic illustration of this SEI layer formation is displayed in Figure 2e, because the larger FSI^−^ size over PF_6_^−^ and the lower lowest unoccupied molecular orbitals (LUMO) levels than solvents according to theoretical calculations, which effectively prevent SEI from cointercalation damage, thus leading to superior cycling stability. Similarly, Mai and co‐workers^[^ ^43^ ^]^ also investigated the effect of 1 m KFSI/EC:propylene carbonate (PC) and 1 m KPF_6_/EC:PC electrolytes on the formation of SEI layer. They proved that NiCo_2.5_S_4_ microrod wrapped in reduced graphene oxide (denoted as NCS@RGO) has a thinner SEI layer in KFSI/EC:PC than that in KPF_6_/EC:PC (Figure 2f,g), and this SEI layer features an excellent mechanical property (Figure 2h). Such a thin and stable SEI film presents lower charge transfer impedance, thus ensuing fast reaction kinetics, as evidenced by electrochemical impedance spectroscopy (EIS) tests. As a result, superior electrochemical performances are achieved in terms of rate capacity (402 mAh g^−1^ at 2000 mA g^−1^), cycling stability (495 mAh g^−1^ at 200 mA g^−1^ over 1900 cycles), and high initial Coulombic efficiency (ICE: 78%). Since the higher stability of PF_6_^−^ against the reduction/decomposition than that of FSI^−^, a KF‐rich SEI layer with high stability can be well formed in KFSI/EC:DEC, which is more favorable for maintaining interfacial chemical or electrochemical reaction. So, when choosing KFSI over KPF_6_ dissolved in EC/DEC as electrolyte, both the capacity retention and CE of commercial micrometric MoS_2_ are largely improved.^[^ ^44^ ^]^ As we all know, in situ characterization can provide real‐time monitoring for exploring accurate physical structure and chemical component of SEI film. With the help of Fourier transform infrared (FTIR) spectroscopic mapping, Guo and co‐workers^[^ ^45^ ^]^ found that SEI layer is formed on the electrode surface after the 1st cycle for both KPF_6_/EC:DEC and KFSI/EC:DEC electrolytes, but the formed SEI in KFSI/EC:DEC is more homogeneous and thinner than that in KPF_6_ electrolyte during cycling. All these together illustrate that KFSI salt can effectively contribute to forming a stable and even SEI layer, which not only benefits the charge transfer, but also exhibits great passivation ability and excellent durability, giving rise to improved capacity and cycle performance. Concretely, the favorable effect of KFSI electrolyte on SEI can be mainly ascribed to its lower reduction stability, that is, the preferential decomposition of KFSI in comparison with KPF_6_.^[^ ^31^ ^]^ Guo and co‐workers^[^ ^46^ ^]^ also demonstrated that the thickness of SEI layer formed on the Bi/reduced graphene oxide (Bi/RGO) surface is similar in both KPF_6_ and KFSI salts after two cycles, as shown in **Figure** **3**a,d, although an obvious difference is observed as the cycle proceeds (Figure 3b,c,e,f). After five cycles, they detected that the SEI layer in KPF_6_ salts is broken due to the structure pulverization of Bi particles (Figure 3b). Accordingly, the new Bi surface is exposed to the electrolyte, leading to the SEI layers with a thickness of up to 10–40 nm ultimately formed after 10 cycles (Figure 3c). In sharp contrast, regardless of thickness or structural integrity, the SEI layer in KFSI salt always keeps stable from 2 to 10 cycles (Figure 3e,f), which is conducive to ions’ fast transfer through it, as confirmed by the surface potential maps (Figure 3g,h), Meanwhile, the results of XPS (Figure 3i–k) verified that the desirable SEI layer formed in KFSI originates predominantly from FSI^−^ anion, which is significantly different from one in KPF_6_. Therefore, Bi/RGO electrode in KFSI salt delivers a considerable rate capability and cycle performance. In addition, red phosphorus (RP)/C anode materials have also verified that the SEI layer generated by KFSI‐based electrolyte is even and robust, and can considerably stabilize interface, thus realizing more significant interface reaction kinetics.^[^ ^47^ ^]^

In short, an ideal SEI layer is usually required to be more stable and robust for slowing down some undesirable side reactions and maintaining continuous ion transfer. And it has been illustrated that KFSI has a lower reduction stability compared to KPF_6_, namely the preferential decomposition of KFSI during initial cycling. Accordingly, the formed SEI layer in KFSI is more stable and uniform in thickness and chemical composition, which is favorable for reducing the continuous consumption of electrolyte active ions. Based on this, excellent interface performances, such as fast ion transfer, improved charge accumulation, and passivation abilities, are achieved. Resultantly, the corresponding electrochemical performances in terms of capacity and cycling life are also greatly enhanced as expected.

### 2.2 Solvent Species

**Figure 4:**
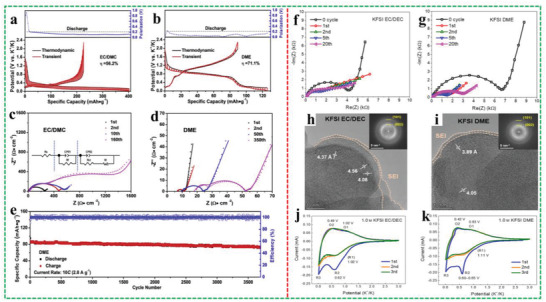
GITT curves and voltage polarization in discharge process a) in EC/DMC‐based electrolyte and b) in DME‐based electrolyte. Electrochemical impedance spectroscopy for graphite at discharge state of 0.01 V in c) EC/DMC‐based electrolyte and d) DME‐based electrolyte. e) Long cycle performance at 10 C in DME‐based electrolyte. Reproduced with permission.^[^ ^56^ ^]^ Copyright 2019, Elsevier Ltd. EIS results for K//HC cells with electrolytes of f) KFSI EC/DEC and g) KFSI DME at fully charged state at the initial and 1st, 2nd, 5th, and 20th cycles. HRTEM images of LHC electrodes after 5 cycles (inset for the corresponding selected area electron diffraction pattern) in h) 1.0 m KFSI EC/DEC and i) 1.0 m KFSI DME. CV profiles of K//LHC half‐cells with j) 1.0 m KFSI EC/DEC and k) 1.0 m KFSI DME as the electrolytes. Reproduced with permission.^[^ ^41^ ^]^ Copyright 2022, Elsevier Ltd.

Solvent, as one of the major components of electrolyte, has a substantial effect on the chemical or electrochemical interfacial by modifying the formation of SEI layer.^[^ ^30^, ^48^, ^49^ ^]^ It has been demonstrated that the use of organic solvent in PIBs is similar to that of LIBs and sodium‐ion batteries (SIBs), mainly including esters (EC, PC, dimethyl carbonate (DMC), DEC, etc.) and ethers (ethylene glycol dimethyl ether (DME) and diethylene glycol dimethyl ether (DEGDME)).^[^ ^35^ ^]^ Additionally, the most important properties of these solvents are listed in **Table** **2**, and it is well accepted that LUMO and the highest occupied molecular orbital (HOMO) are closely related to solvents molecular properties,^[^ ^34^, ^50^ ^]^ so they are usually used to roughly evaluate the reductive/oxidative stability of solvents. Although organic solvents EC, DEC, DMC, and PC have similar LUMO and HOMO, recent studies have proved that EC/DEC‐ or EC/DMC‐based electrolytes can be severely decomposed at a low potential compared to EC/PC‐based electrolytes, thus leading to more side reactions related to the formation of unstable and uneven SEI. Therefore, Zhao et al.^[^ ^48^ ^]^ used the electrolyte of KPF_6_ in EC:PC instead of KPF_6_ in EC:DEC or EC:DMC to smoothly improve interfacial stability of K||graphite cells. However, EC/DEC‐based electrolytes are not always unfavorable for SEI, and can also form a highly stable, intact, and robust SEI in some specific cases. Peng et al.^[^ ^51^ ^]^ reported graphite oxide with oxygen‐rich functional groups, and they considerably illustrated that the presence of C═O and COOH groups contributes to forming a inorganic component SEI in EC/DEC electrolyte, as confirmed by in situ FTIR analysis. This desirable SEI film endows smaller SEI resistance (*R*_SEI_), charge transfer resistance (*R*_ct_), and higher ion diffusion coefficient, thus leading to increased reaction dynamic, as evidenced by EIS measurement results. Furthermore, dissolving KPF_6_ into ether solvents, such as DME and DEGDME, is more beneficial for forming thin and robust SEI film by reducing the generation of some side reactions, and as a result good cycling stability and high ICE are successfully achieved in 1 m KPF_6_/DME and DEGDME electrolytes for dipotassium terephthalate, Bi, and SnSb anodes.^[^ ^52^, ^53^, ^54^ ^]^ Furthermore, Lei et al.^[^ ^55^ ^]^ also clarified that an elastic and continuous SEI layer is formed on the Bi particle surface in DME‐based electrolyte, which strongly keeps particles integrated to avoid the loss of active material. By contrast, there are severe pulverization and visible electrode isolations of Bi anode after cycles in EC/PC‐based electrolytes. Particularly, the binding energies between K ions and ether molecules are higher than esters, which can allow the cointercalation of K ions and solvent molecules into graphite anode. Because of the lack of a desolvation process, a fast diffusion kinetics of K^+^–DME complexes and a negligible SEI layer in DME‐based electrolyte are guaranteed. In other words, these cointercalation behaviors will not block the SEI layer, and at the same time, thermodynamic stable phases with a small volume expansion are also formed. Wang et al.^[^ ^56^ ^]^ demonstrated, through galvanostatic intermittent titration technique (GITT) (**Figure** **4**a,b), that K is comparatively easier to intercalate into graphite in DME. And DME‐based electrolyte exhibits a smaller charge transfer impedance compared to EC/DMC‐based electrolyte (Figure 4c,d), thus achieving an excellent interfacial reaction kinetics at a high current density (Figure 4e). As for the in‐depth characterization of the cointercalation mechanism and formation of stage graphite intercalation compound, the most commonly used technologies are in situ Raman spectroscopy, X­ray diffraction and ex situ FTIR spectroscopy measurements, first principle calculations.^[^ ^56^, ^57^ ^]^ In addition to DME‐based electrolyte, DEGDME‐based electrolyte in a graphite anode also generates a thinner SEI layer (0.5–1.5 nm) in comparison with EC/DEC electrolyte.^[^ ^58^ ^]^ Such a thin electrolyte/electrode interface enables the continuous transport of K ions, and functions as a passivation layer to prevent electrode structure from collapsing.

**Table 2:** Physical and chemical properties of the reported solvents for PIB electrolytes^[^ ^34^, ^50^ ^]^

Solvent	Melting point [°C]	Boiling point [°C]	Flash point [°C]	Density 25 °C [g mL^−1^]	Viscosity at 25 °C [cP]	Dielectric constant at 25 °C
EC	36.4	248	160	1.32	2.1	0.0 ± 0.5
PC	−48.8	242	132	1.20	2.5	0.0 ± 0.4
DMC	0.5	91	18	1.07	0.6	56 ± 0.1
DEC	−43	126	31	0.98	0.8	11.5 ± 0.2
DME	−69	84	0	0.87	0.5	80.6 ± 0.1
DEGDME	−64	162	57	0.94	1.1	3.2 ± 0.2

However, it is found that the DME‐based electrolyte with KPF_6_ salt presents more side reactions in hard carbon (HC) anode, giving rise to unstable SEI layer. Furthermore, this undesirable SEI is unfavorable for maintaining a high interfacial stability, and accelerating ion transfer, thus leading to fast capacity decay and unsatisfactory rate capability. The effect of KFSI in EC/DEC electrolyte and KFSI in DME electrolyte on the SEI film is also investigated in HC anode by Liu and co‐workers.^[^ ^41^ ^]^ They discovered that the *R*_ct_ of both KFSI EC/DEC and KFSI DME electrolytes decreases obviously after the generation of SEI layer at the 1st cycle (Figure 4f,g), and they ascribed this phenomenon to the formed SEI membrane which effectively improves the charge transfer kinetics. Besides, KFSI DME electrolyte can form a relatively thin SEI layer (Figure 4h,i), and no oxidation peaks are observed at high voltages, indicating that FSI^−^ has an “inhibitory effect” for DME decomposition at 3 V (Figure 4j,k). But the decomposition of FSI^−^, EC, and DEC are more beneficial for the formation of SEI layer, while only FSI^−^ benefits the SEI in KFSI DME. Noticeably, the SEI induced by the decomposition of FSI^−^ in KFSI EC/DEC has excellent elasticity and stability, which can separate the electrolytes from HC anode, hampering the occurrence of side reactions. And FSI^−^ decomposes before EC so that K ions have enough EC to complete the solvation; as a result, solvated K ions not only can fast transfer charge around SEI layer, but also protect SEI from being damaged by bare K ions, thus substantially enhancing the interfacial kinetics. On the contrary, when KFSI DME electrolyte is applied in the other anodes for PIBs, it delivers a beneficial influence on the SEI in comparison with KFSI EC/DEC. This can be explained by the fact that the main components of formed SEI layer are inorganic salts derived from the reduction of KFSI rather than DME. For example, Li and co‐workers^[^ ^59^ ^]^ intensively investigated the evolution of SEI layer in 1 m KFSI DME and 1 m KFSI EC/DEC electrolyte for three‐dimensional (3D) foam‐ like graphenic carbon scaffold incorporated with FeP nanoparticles (FeP@FGCS) electrode by performing a series of ex situ XPS tests. They confirmed that the main components of SEI layer formed in 1 m KFSI DME are inorganic salts, while the one in 1 m KFSI EC/DEC is primarily composed of unstable organic components derived from the EC and DEC. It is well accepted that the inorganic SEI species are more stable, and simultaneously can effectively avoid direct contact between the active materials and solvent molecules, thus ensuring stabilized electrode/electrolyte interface, which favors a better conductivity and ion diffusion capability. As expected, the FeP@FGCS electrode in 1 m KFSI DME system manifests a prominent rate performance (221 mAh g^−1^ at 2000 mA g^−1^) along with an ultralong cycle lifespan over 3000 cycles with a capacity of 183 mAh g^−1^.^[^ ^59^ ^]^ Similarly, Mao and co‐workers^[^ ^60^ ^]^ compared the difference between 3 m KFSI DME and 3 m KFSI EC/DEC electrolyte on the formation of SEI in SnSb/C anode. The study found that the generated SEI in 3 m KFSI DME is dominated by more inorganic components, thus promoting K‐ion diffusion, and maintaining structure stability of electrode.

As mentioned above, solvents, including esters (EC, PC, DMC, DEC, etc.) and ethers (DME and DEGDME), play a vital role in thickness and chemical composition of SEI species. Due to the higher electrochemical stability and ability to dissolve K salts, ester solvents are widely reported in previous reports, but numerous researches have proved that DME or DEGDME is more conducive to the formation of a desirable SEI layer in most anodes. Particularly, it is worth noting that the cointercalation of K ions and solvent molecules into graphite can avoid the sluggish desolvation process in EC/DMC‐based electrolytes, enabling a fast interfacial reaction kinetics. Sometimes, when using KFSI EC/DEC electrolyte, due to the synergistic contribution of inorganic anions (FSI^−^) and organic solvents (EC and DEC), the formed SEI is more desirable for realizing interfacial chemical properties. Consequently, the final electrochemical performance of anode materials can be significantly elevated, such as capacity, cycling life, rate capability.

### 2.3 Electrolyte Concentration and Additive Species

**Figure 5:**
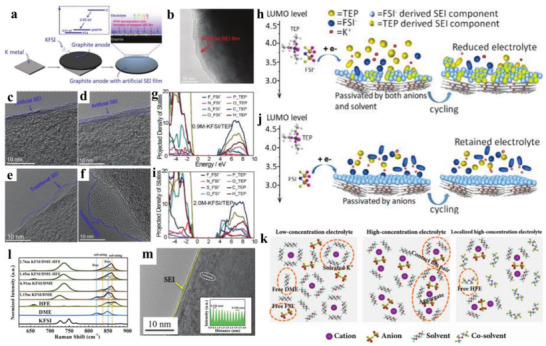
a) Process that spontaneously forms an artificial SEI on a graphite anode in a KFSI–DME electrolyte. b) The HRTEM image of an artificial SEI film formed on a graphite anode. c,d) The HRTEM image of artificial SEI film and e,f) traditional SEI film on graphite anode after 5 cycles (c, e) and 50 cycles (d, f). Reproduced under the terms of the Creative Commons CC‐BY license.^[^ ^39^ ^]^ Copyright 2021, The Authors. Published by Wiley‐VCH. g) 0.9 m KFSI/TEP and i) 2 m KFSI/TEP electrolytes from density functional theory molecular dynamics (DFT‐MD). Schematic illustration of SEI formation under h) 0.9 m KFSI/TEP passivation and j) 2 m KFSI/TEP passivation. Reproduced with permission.^[^ ^65^ ^]^ Copyright 2019, Wiley‐VCH. k) Schematic illustrations of solution structures of low concentration electrolyte (LCE), HCE, and LHCE based on KFSI/DME with/without HFE. l) Raman spectra of KFSI/DME and KFSI/DME–HFE electrolytes. m) TEM image on the cycled graphite electrode after 20 cycles with 2.76 m KFSI/DME–HFE electrolyte. Reproduced with permission.^[^ ^70^ ^]^ Copyright 2019, Wiley‐VCH.

Generally, in order to balance the ionic conductivity, viscosity, and solubility of K salts, electrolytes for PIBs contain less than 1.5 m K salts.^[^ ^31^, ^32^ ^]^ These diluted electrolytes are the most common choice for PIBs, but the unsatisfactory interfacial properties of formed SEI usually give rise to poor electrochemical performance of K half‐cells. From the point of view of solvation structure, increasing the salt concentration may be an effective strategy to enhance the stability of SEI, this is because the interaction of cation–solvent or cation–anion is obviously improved, and the content of free‐state solvent molecules will be suppressed,^[^ ^61^, ^62^ ^]^ simultaneously. Under this context, the development of high‐concentration electrolyte has attracted great attentions for enhancing battery performance, especially interfacial behaviors. To the best of our knowledge, graphite can effectively store potassium by forming K‐intercalation compounds accompanying with a theoretical specific capacity of 279 mAh g^−1^. In spite of this, the unstable interfacial reaction induced by the repeated formation of SEI in organic electrolytes inhibits its further application. Recently, Lu and co‐workers^[^ ^39^ ^]^ developed an artificial SEI layer on the graphite anode using a high concentration electrolyte (3 m KFSI–DME), and the construction process and microstructure of artificial SEI layer are shown in **Figure** **5**a,b, respectively. They found that this artificial SEI layer is ultrathin, uniform, dense, and stable (Figure 5c,d), and markedly outperforms traditional SEI film that is discontinuous and nondense (Figure 5e,f); as a result, such a desirable SEI tends to prevent the electrolyte from further decomposition during potassiation/depotassiation, and therefore realizes a long cycle performance over 1000 cycles, a high ICE of 93%, and a capacity retention as high as 100%. Analogously, Lu and co‐workers^[^ ^63^ ^]^ selected high concentration electrolyte (3 m KFSI/DME) to form a highly stable and effective SEI layer on the carbonaceous material surface. Through the thermodynamic analysis, they proved that the SEI, featured by inorganic species, mainly comes from the decomposition of KFSI. Such an inorganic SEI enables a stable interfacial performance, thus leading to an ultralong cycle lifespan (over 14 000 cycles at 2000 mA g^−1^). The beneficial effect of 3 m KFSI–DME electrolyte on SEI layer also enables a good interfacial chemical performance for *α*‐NiS+RGO electrode,^[^ ^64^ ^]^ thus endowing a superior ICE of 87.9%.

Another interesting finding is that a significant amount of free solvent molecules in the diluted electrolyte will make both solvent and salt decompose, which has been evidenced by theoretical and experimental results.^[^ ^65^ ^]^ The continuous consumption of both solvent and salt in a low concentration electrolyte, such as 0.9 m KFSI/triethyl phosphate (TEP), inevitably forms a nonuniform and nonstable SEI, thus causing capacity decay (Figure 5g,h). However, a long cyclability can be achieved by using a high concentration electrolyte (3 m KFSI/TEP), which is mainly because a single decomposition source (salt) has a higher ability to generate a uniform and dense SEI film (Figure 5i,j). It should be pointed out that the as‐obtained SEI layer derived from salt is often composed of inorganic components, thus sufficient interfacial contact between these K‐containing inorganic compounds promotes K‐ion migration along their boundaries. Not only that, when using ester solvent, a robust KF‐rich SEI layer on Sb@carbon sphere network (CSN) anode is still obtained by coupling 4 m KTFSI/EC+DEC.^[^ ^66^ ^]^ After the 100 charge/discharge cycles, electron‐involved reaction impedance exhibits a slight increase in tendency, which is closely related to a stable reaction interface enabled by robust SEI. Despite these progresses, some challenges in high concentration electrolyte (HCE) system cannot be ignored, that is, high viscosity, low ionic conductivity, and the increased cost, all of which still hinder its realistic applications. For example, high viscosity usually causes a worse wettability of electrolyte, leading to increased initial charge transfer resistance in comparison with a diluted electrolyte, and low ionic conductivity is unfavorable for achieving a fast reaction kinetics. To better solve these bottlenecks, the introduction of cosolvent into HCE to form a localized high‐concentration electrolyte (LHCE) has been proven to be an effective method, and the introduced cosolvent does not participate in the solvation process.^[^ ^67^, ^68^, ^69^ ^]^ In view of this, Wu and co‐workers^[^ ^70^ ^]^ utilized a highly fluorinated ether (HFE) as cost‐effective cosolvent to dilute the concentrated KFSI/DME electrolyte. The as‐obtained LHCE, namely 2.76 mol kg^−1^ KFSI/DME–HFE, is expected to break the reinforced 3D network while retaining the local coordination structure of cations in a LHCE (Figure 5k). Besides, the presence of HFE has little effect on K^+^ solvation structure, as evidenced by Raman spectra in Figure 5l. Based on the above analysis, they concluded that the HFE only lowers the concentration of KFSI/DME electrolyte without any other effects; accordingly, an amorphous SEI film with the thickness of less than 1 nm is formed on the graphite surface (Figure 5m), and with the help of this robust SEI film, graphite anode still preserves its compact and layered structure even after long cycling. Benefiting from improved ionic conductivity, ultrathin and stable SEI in LHCE, graphite anode delivers a capacity of 200 mAh g^−1^ with a high Coulombic efficiency of 99.5%. Another research on the beneficial roles of LHCE (KFSI/TMP/HFE = 1:1.7:2 by mol, where TMP is trimethyl phosphate) in building robust SEI layer is also reported,^[^ ^71^ ^]^ and the as‐obtained materials with this SEI display an exceptional reversible capacity (618 mAh g^−1^ under 100 mA g^−1^), and outstanding long‐term cycling stability. Through optimizing the proportion of KFSI salt and TMP solvent instead of introducing cosolvent, Liu et al.^[^ ^72^ ^]^ prepared a moderate‐concentration electrolyte (the molar ratio of TMP and KFSI in 3:8) to considerably realize an excellent cycling stability in graphite anode as a result of the improved interfacial behavior enabled by nearly 100% solvation of TMP molecules with K^+^ cations and the formation of FSI^−^‐derived F‐rich SEI film.

**Figure 6:**
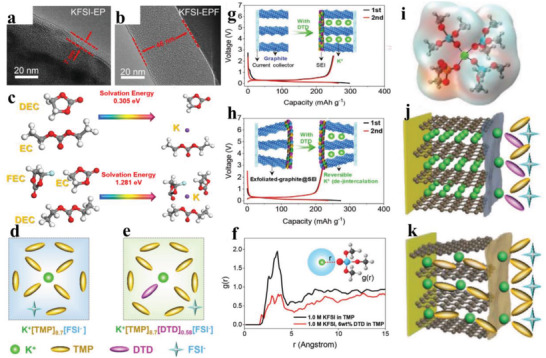
TEM image of NCS@RGO‐2 with a) KFSI–EP and b) KFSI–EPF electrolytes, respectively. Reproduced with permission.^[^ ^43^ ^]^ Copyright 2019, Wiley‐VCH. c) Solvation energies estimated from the binding energy of the K^+^ (Y) clusters. Reproduced with permission.^[^ ^45^ ^]^ Copyright 2018, Elsevier Ltd. d,e) Schematic illustration of molecular interaction between the solvent (TMP), the solute of KFSI, and DTD additives. f) Radial distribution function (RDF) of K^+^ to the oxygen in TMP without and with DTD additives. Reversible K^+^ (de)intercalation g) within graphite electrode and h) within exfoliated graphite@SEI in electrolyte with 6 wt% DTD additives, respectively. i) Snapshot of the first solvation shell of K^+^ with DTD additives. Different interfacial models and electrochemical behaviors of graphite using different electrolytes of 1.0 m KFSI in TMP j) with and k) without DTD additives. Reproduced with permission.^[^ ^80^ ^]^ Copyright 2020, Wiley‐VCH.

Sometimes, in order to modify SEI layer to better stabilize electrode/electrolyte interface, some additives need to be incorporated into electrolyte system, and related reports can be easily observed in LIBs and SIBs.^[^ ^73^, ^74^ ^]^ As for PIBs, it has been reported that the Coulombic efficiency and cycling capability of Prussian blue analog cathodes can be significantly improved by adding fluoroethylene carbonate (FEC) into EC/DEC electrolytes.^[^ ^75^ ^]^ Unfortunately, FEC additive has an adverse effect on SEI property for PIB anode, which is distinctly different from previous works on LIBs and SIBs.^[^ ^76^, ^77^ ^]^ This phenomenon is likely to associate with the continuous consumption of FEC upon cycling, due to its inferior reductive stability caused by low LUMO,^[^ ^31^, ^34^ ^]^ which inevitably brings out excessive side reactions and hence results in undesirable SEI layer. For instance, Mai and co‐workers^[^ ^43^ ^]^ found that NCS@RGO‐2 electrode manifests a faster capacity fading in 1 m KFSI/EC+PC with 5% FEC (KFSI–EPF) than 1 m KFSI/EC+PC (KFSI–EP), and through further characterizations, they ascribed this phenomenon to the formation of thick SEI layers on the NCS@RGO‐2 surface with the introduction of FEC (**Figure** **6**a,b). Similar phenomenon is also demonstrated by Guo and co‐workers,^[^ ^45^ ^]^ they verified that K‐ion diffusion and desolvation process are more difficult in the electrolyte with 5 wt% FEC (Figure 6c), thus unfavoring the rate performance. Without exception, owing to the severe side reactions in electrolytes with FEC,^[^ ^78^ ^]^ Sn_4_P_3_ anode has similar electrochemical behaviors with NCS@RGO‐2 and GeP_5_, that is, poor cycle life with fast capacity decay. Compared to FEC, ethylene sulfate (DTD, C_2_H_4_O_4_S) seems to be a good additive because of its capability to form robust SEI and strong coordination architecture (i.e., S═O) in electrolyte.^[^ ^79^ ^]^ Ming and co‐workers^[^ ^80^ ^]^ demonstrated that the K^+^ solvation structure exhibits an obvious change via adding 6 wt% DTD into 1.0 m KFSI in TMP (Figure 6d,e), and which is further confirmed through simulation results (Figure 6f). When they applied this electrolyte with DTD to graphite anode, K^+^–solvent coinsertion is well suppressed by the formed robust SEI film (Figure 6g). They also signified that the exfoliated graphite@SEI can store K ions reversibly without the K^+^–solvent coinsertion, due to the strong protective effects of SEI membrane, which is supported by Figure 6h. Besides, the reduced interaction of K^+^–TMP and K^+^–FSI^−^, as evidenced by Figure 6i–k, is also responsible for a stable SEI layer related to continuous interfacial ion transfer. All these results illustrate that robust SEI well prevents graphite from exfoliating by delivering an excellent rate capacity (228 mAh g^−1^ at 2.4 C, 1 C = 280 mA g^−1^), and a prominent cycle capability at 100 mA g^−1^ over 100 cycles with 272 mAh g^−1^ capacity. In addition to FEC and DTD additives, Matsumoto and co‐workers^[^ ^81^ ^]^ successfully established a robust SEI with KF‐rich species on graphite surface by employing potassium difluorophosphate (KDFP) additive, which significantly improves interface transport kinetics, and hence increases cycle life, Coulombic efficiency, and a high capacity retention of 76.8% over 400 cycles.

In summary, high concentration electrolyte can effectively reduce the amount of free solvent molecules in electrolyte, so that only the potassium salt is decomposed rather than the codecomposition of salt and solvent, leading to the formation of an inorganic SEI featured by stable and dense. Despite that, high viscosity and relatively low ionic conductivity caused by high concentration electrolyte cannot be ignored, and these obstacles can be well solved through the introduction of cosolvent to form localized high‐concentration electrolyte. Particularly, the introduced cosolvent only lowers electrolyte concentration without any other effects, thereby an inorganic SEI still is retained. Considering the widespread application of FEC additive in LIBs and SIBs, it is also investigated in PIBs but the related results are unsatisfactory, this is because more side reactions are formed during cycling. In contrast to FEC, DTD and KDFP additives can generate a roust SEI to stabilize and improve electrode/electrolyte interface, thus distinctly promoting the realization of superior cycling stability and rate capability.

### 2.4 Binders

**Figure 7:**
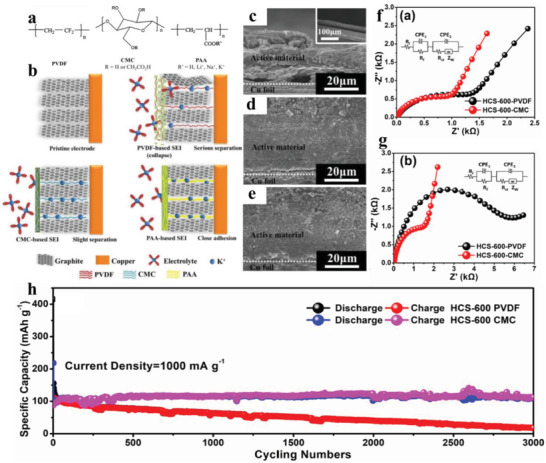
a) Molecular structures of the three kinds of polymeric binders of PVDF, CMC, and PAA. b) Schematic illustrations of the plausible interface structure in different binder systems. Cross‐sectional SEM images of the graphite composite electrodes c) 10% PVDF, d) 10% CMCNa, and e) 10% PAANa. Reproduced with permission.^[^ ^92^ ^]^ Copyright 2018, Springer. EIS spectra of HCS‐600 f) before and g) after 50 cycles. h) Long‐term cycling performance at 50 and 1000 mA g^−1^. Reproduced with permission.^[^ ^82^ ^]^ Copyright 2019, American Chemical Society.

It is well‐known that substantial volume fluctuation during repeated potassiation/depotassiation will inevitably cause electrode structure collapse or pulverization, resulting in the active particles being exfoliated from the current collector, and the new active surfaces are exposed to electrolyte.^[^ ^82^, ^83^, ^84^ ^]^ As binders, their main function is first to form covalent bonds between active materials and themselves, and the formed covalent bonds can effectively hamper the pulverization of active particles;^[^ ^85^ ^]^ subsequently, the cross‐linked polymer characteristic of binders will well separate the electrolyte from the active particles by forming a protective layer on the electrode, which is conducive to suppress the overgrowth of SEI layer; besides, the strong interactions between active particles and binders considerably restrict the irreversible movement of active particles, thus the formed SEI films will not be destroyed.^[^ ^86^ ^]^ Based on abovementioned analysis, apart from overall electrolyte systems, binders also play a vital role in determining the SEI properties in terms of thickness, stability, and uniformity. At present, commercial polyvinylidene fluoride (PVDF) is still the most commonly used binder in PIBs,^[^ ^8^, ^87^ ^]^ although many binder‐free electrodes have been reported.^[^ ^88^, ^89^ ^]^ Notably, hydrosoluble sodium carboxymethyl cellulose (CMCNa) and sodium polyacrylate (PAANa), as alternatives to PVDF, have attracted enormous attention because of their strong tolerating and adhesion abilities.^[^ ^90^, ^91^ ^]^ Komaba et al.^[^ ^91^ ^]^ found that CMCNa and PAANa tend to form a robust SEI to modify electrode/electrolyte interface, thus exhibiting higher ICEs compared to traditional PVDF binder. Similar work is also performed by Zhuang and co‐workers (**Figure** **7**a),^[^ ^92^ ^]^ they intensively analyzed the interface structure of graphite anode in three types of binders (Figure 7b). Through Figure 7c–e, it can be clearly seen that PAANa composite electrode tightly adhered to the current collector, which can effectively prevent the repeated formation of SEI layer, thus endowing a stable and continuous interfacial ion transfer behavior. Not only graphite anode, the essential influence of PAANa binder on SEI films is also investigated in transition metal oxides. Liu et al.^[^ ^93^ ^]^ discovered that three‐dimensional N‐doped porous graphene framework decorated with Fe_3_O_4_ nanoparticles (Fe_3_O_4_/3DNPGF) electrode with PAANa binder achieves a better interfacial kinetics than its PVDF binder counterpart, such as fast charge transfer capability and improved structure stability. Through an in‐depth analysis, they indicated that this difference is primarily due to the PAANa forming a uniform and stable SEI layer on the electrode surface.

Because of the lack of the defluorination effect and superior coatability of carboxymethyl cellulose (CMC) binders, the excessive depletion of the electrolyte can be effectively reduced, thus ensuring the formation of uniform SEI layer. Mai and co‐workers systematically compared the effect of CMC and PVDF on the SEI layer in porous hollow carbon sub‐microsphere anodes (HCS‐600).^[^ ^82^ ^]^ A comparison of the scan‐ ning electron microscopy (SEM) images after 50 cycles intuitively unravels that HCS‐600 with PVDF binder has a noticeable crack, while CMC‐binder‐based electrode presents a complete and dense surface. EIS tests before and after 50 cycles (Figure 7f,g) reveal that HCS‐600 with CMC binder has more excellent charge transfer kinetics during cycling. Through XPS characterization results, they proved that CMC binder can form a uniform passivation layer on the surface of active materials, as the pre‐SEI layer, it can greatly maintain structural integrity of electrode, thereby avoiding the exposure of fresh active surface. Resultantly, reversible capacity is increased from 89 mAh g^−1^ for PVDF‐based electrode to 211 mAh g^−1^ for CMC‐based electrode; even at 1000 mA g^−1^ (Figure 7h), HCS‐600 with CMC binder retains 111 mAh g^−1^ after 3000 cycles, which is extremely far above 18 mAh g^−1^ in PVDF binder. Different from Mai and co‐workers, Guo and co‐workers^[^ ^60^ ^]^ studied the role in interfacial reaction played by different types of CMC binders, namely CMC, CMC+styrene‐butadiene (SBR), CMC+/cellulose acetate (CA), and CMC+ polyacrylic acid (PAA). They demonstrated that only CMC+PAA‐based electrode displays a smooth surface without obvious cracks, while the electrode surfaces in other three binders have varying degrees of cracks. These results suggest that CMC+PAA binder has the most superior ability to tolerate the volume expansions, thus preventing the repeated formation of SEI. Naturally, the electrode with CMC+PAA exhibits excellent electrochemical kinetics, as evidenced by the rate capacities at high current densities of 500 and 1000 mA g^−1^, because the thin and stable SEI allows ions’ fast transport; however, due to the thick and unstable SEI, it is difficult for electrodes in CMC, CMC+SBR, and CMC+CA binders to obtain satisfactory interfacial dynamics as CMC+PAA.

To briefly summarize this section on binders, what we want to emphasize is that the binder can not only fix the active particles on the current collector, but also significantly affect the performance of SEI. Although commercial PVDF binders are still widely applied in PIBs up to now, they cannot well maintain the structure stability during repeated potassiation/depotassiation, thus easily causing the overgrowth of SEI layer. In contrast to that, CMCNa and PAANa binders are more reliable to accommodate huge volume fluctuation of electrode, and construct stable SEI, endowing improved interfacial diffusion capability. Nevertheless, the researches on CMCNa and PAANa binders in PIBs are relatively lacking, so increasing attention needs to be attached. Moreover, in‐depth characterization and analysis are extremely important for revealing the intercorrelation between binders and SEI properties, thus theoretical calculations and other assisted tools may be a good choice to improve research efficiency.

### 2.5 Electrodes

**Figure 8:**
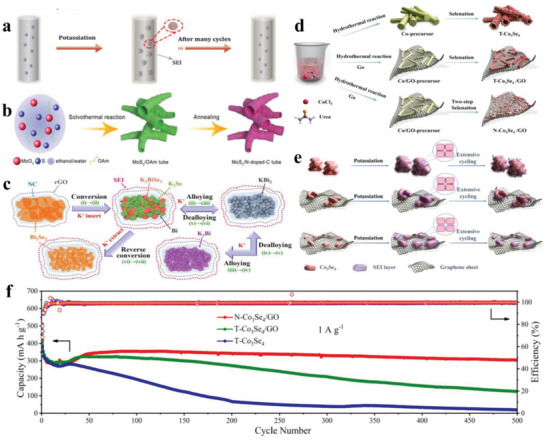
a) Schematic illustration of the morphology changes and SEI formation upon electrochemical processes. Reproduced with permission.^[^ ^98^ ^]^ Copyright 2019, American Chemical Society. b) Synthesis process of the porous MoS_2_/N‐doped‐C tube. Reproduced with permission.^[^ ^99^ ^]^ Copyright 2018, Wiley‐VCH. c) Schematic view of the proposed electrochemical mechanism during the charge/discharge process. Reproduced with permission.^[^ ^100^ ^]^ Copyright 2020, Wiley‐VCH. d) Schematic illustration of the synthesis process and e) morphology evolutions of T‐Co_3_Se_4_, T‐Co_3_Se_4_/GO, and N‐Co_3_Se_4_/GO, during the potassiation process. f) Long‐term cycle stability of three electrodes at 1000 mA g^−1^ over 500 cycles. Reproduced with permission.^[^ ^103^ ^]^ Copyright 2020, American Chemical Society.

In addition to the optimization of electrolyte systems and binders, developing novel electrode with unique architecture is an effective strategy for developing stable SEI, this is because K^+^ with larger ionic size inserts into electrode usually causes huge volume expansion.^[^ ^94^, ^95^, ^96^ ^]^ Once these volume expansions cannot be well alleviated, the formed SEI on the electrode surface will be inevitably destroyed, which easily leads to poor interfacial transfer kinetics, accompanied by inferior rate and cycle capabilities. For example, Bi can alloy with three K to form K_3_Bi accompanied with a high volumetric theoretical capacity of above 700 mAh cm^−3^,^[^ ^97^ ^]^ but its severe volume variation will cause a large number of newly exposed active surfaces, thus leading to continuous generation of SEI layer. Resultantly, charge transfer ability is significantly impeded, and further induces fast capacity fading. Lu and co‐workers^[^ ^98^ ^]^ reported a novel ultrathin carbon film@carbon nanorods@Bi nanoparticles (UCF@CNs@BiNs), and they demonstrated that the presence of UCF@CN matrix not only accommodates the volume variation of Bi nanoparticles, but also promotes the formation of SEI on external carbon film rather than individual Bi nanoparticles, successfully avoiding the fracture of SEI even after repeated cycling (**Figure** **8**a). Thus, this UCF@CNs@BiN electrode delivers a high capacity of ≈327.0 mA g^−1^ and a prolonged cycle life over 700 cycles. Like the above design strategy, a bamboo‐like MoS_2_/N‐doped‐C (Figure 8b) is presented,^[^ ^99^ ^]^ and this N‐doped‐C configuration inhibits the continuous growth of SEI in the interior of the particles by alleviating volume change of MoS_2_ during K‐ion insertion/extraction process. Accordingly, MoS_2_/N‐doped‐C exhibits a satisfactory reversible capacity of 151 mAh g^−1^ at 500 mA g^−1^, and a superb rate performance of 131 mAh g^−1^ at 2000 mA g^−1^, while pure MoS_2_ electrode shows sharp capacity fading after 50 cycles due to its unstable SEI induced by substantial structure pulverization. Wang and co‐workers^[^ ^100^ ^]^ developed a Bi_2_Se@NC@RGO composite, in which they proved that strong C—O—Bi bonding can provide superior electrode integrity, and the NC coating layer and RGO release well stress derived from large volume variation. Therefore, regardless of the subsequent conversion or alloying reaction, the formed SEI on the Bi_2_Se@NC@RGO surface is always well preserved (Figure 8c). As a result, Bi_2_Se@NC@RGO fulfills a good cycling stability and an excellent rate capacity of 101.6 mAh g^−1^ at 5000 mA g^−1^. Besides, multicore–shell Fe_2_N–carbon nanofiber electrode is also reported,^[^ ^101^ ^]^ and this multicore–shell architecture together with the long‐range 1D carbon nanofiber framework is proven to be a buffer zone to accommodation volume expansion/contraction, preventing the continual rupturing and reformation of the SEI film. Even after long cycling, SEI layer on the electrode surface has no obvious change, thus prominent electrochemical performances are obtained. Not only that, Li and co‐workers^[^ ^102^ ^]^ well established a 3D N‐doped graphene framework coupled with Fe_3_C@porous graphite carbon core–shell structures (Fe_3_C@PGC–NGF). Through ex situ high‐resolution transmission electron microscope (HRTEM) observations, they confirmed the reversible formation/dissolution of SEI layer, and further described that the reversible change of SEI layer makes numerous K ions participate in the electrochemical reactions instead of being stored as dead potassium in the electrode. Therefore, when Fe_3_C@PGC–NGF as an anode is evaluated in PIBs, it achieves a high capacity, ICE, and outstanding cycling stability in comparison with pure Fe_3_C without PGC–NGF configurations. In our previous works,^[^ ^23^ ^]^ we used Chinses Paper as raw materials to prepare the carbon fiber film with SiO_2_ and MgO nanoparticles on its surface, and we found that the SiO_2_ and MgO nanoparticles are favorable for forming an even and thin SEI (2–4 nm), as evidenced by a thicker and unstable SEI induced by moving SiO_2_ and MgO nanoparticles. Such a thin SEI layer is thick enough to prevent the electron penetration and allows unhindered K‐ion intercalation/deintercalation, simultaneously. Moreover, the interface between active material and electrolyte can well be stabilized, thus leading to a fast charge transfer ability. All these together ensure a considerable cycle of over 500 cycles at 500 mA g^−1^ along with a high‐rate performance of 177.3 mAh g^−1^ at 2500 mA g^−1^.

Introducing carbon matrix and simultaneously optimizing host materials structure also play a key role in maintaining SEI stability. Wang and co‐workers^[^ ^103^ ^]^ not only used graphene to accommodate the volume expansion of Co_3_Se_4_ (N‐Co_3_Se_4_/GO), but also found that Co_3_Se_4_ nanocrystallites are more uniformly distributed in graphene matrix compared to Co_3_Se_4_ nanotubes (T‐Co_3_Se_4_/GO) (Figure 8d), which gives rise to reduced compressive stress caused by the mutual extrusion of nanocrystallites. Based on the synergistic effect of graphene substrate and nanocrystallite structure, the formed SEI layer can be well maintained during repeated cycling (Figure 8e). These improved SEI properties make N‐Co_3_Se_4_/GO electrode a superior electrochemical performance in terms of high capacity of 451.5 mAh g^−1^, rate capability, and cyclability at 1000 mA g^−1^ (Figure 8f). Another study worth mentioning is that nitrogen‐doped carbon‐coated Cu_2_S hollow nanocubes (Cu_2_S@NC),^[^ ^104^ ^]^ in which inner cavity in hollow nanocubes can relieve volume expansion during cycling, ensuring structural integrity; while surface‐coated nitrogen‐doped carbon layer can avoid direct contacting between electrode/electrolyte, reducing continuous decomposition of the electrolyte to form a thick and unstable SEI. In contrast to that, due to the lack of hollow structure and carbon coating layer, the pure Cu_2_S blocks exhibit an unstable SEI layer on their surface. These effects of different structures between Cu_2_S@NC and pure Cu_2_S blocks on SEI properties are further reflected by electrochemical performance, such as both a high‐rate capacity of 257 mAh g^−1^ at 6000 mA g^−1^ and a long cycle life over 1200 cycles at 1000 mA g^−1^ are observed in Cu_2_S@NC, but it is difficult for pure Cu_2_S blocks to reach a good electrochemical performance at the same condition.

Based on the above considerations, designing and synthesizing a novel electrode with a unique structure, such as hollow structure and core–shell structure, can greatly relieve huge volume variation during K‐ion intercalation/deintercalation into/from electrode via offering sufficient buffer space, thus avoiding continual rupturing and formation of SEI layer. Besides, introducing carbon coating layer can avoid direct contacting between electrode/electrolyte, which greatly reduces the consumption of electrolyte caused by side reactions, favoring a desirable SEI. As a result, the electrode/electrolyte interface is well stabilized, which benefits the battery performances including capacity, rate, and cycling capability.

## 3 Research on Solid/Solid Interface

During the charge storage process, K ions first pass through SEI and then arrive at the surface of electrode, and finally these K ions are stored in the crystal structure. Furthermore, the migration of K ions in electrode usually involves solid/solid interface behavior, which is easily encountered by high transport resistance and low electrochemical reaction, thus causing low‐rate capacity. In this context, controlling interfacial transport to improve diffusion rate of K ions between crystals or inner electrode is of great significance. Concretely, the introduction of heterostructure and expanding interlayer spacing have triggered increasing interest, in which the former, featured by built‐in electric field effect,^[^ ^105^, ^106^ ^]^ can form heterointerface that benefits a rapid diffusion of ion and charge; the latter lowers diffusion barrier of K ions with a larger ionic size within electrode.^[^ ^107^ ^]^ So, in this section, we mainly focus on the in‐depth analysis of K‐ion migration behaviors based on heterointerface and expanded interspacing, and some other effect factors are also discussed briefly.

### 3.1 Heterointerface

**Figure 9:**
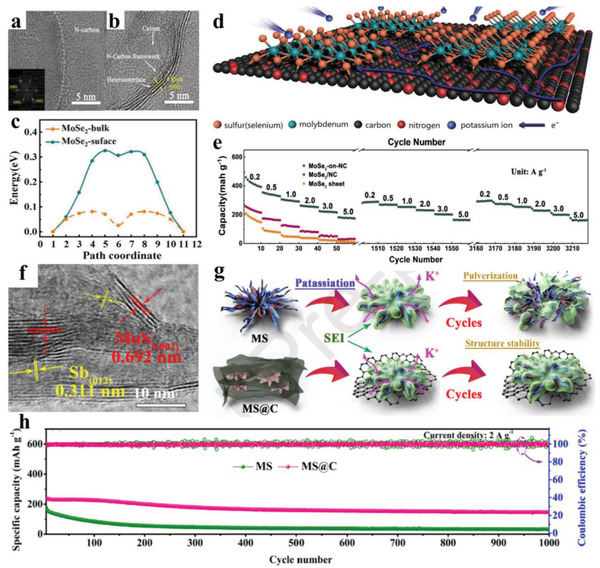
a,b) HRTEM images of heterointerface for MoSe_2_‐on‐NC. c) Migration energy along the two kinds of pathways. d) Schematic illustration of paths for electron conduction/potassium‐ion diffusion on the MoSe_2_‐on‐NC. e) Comparison of rate cycle performance at various current densities. Reproduced with permission.^[^ ^109^ ^]^ Copyright 2020, Wiley‐VCH. f) HRTEM images of MS@C composite. g) Schematic illustration of MS@C and MS composites during long‐term cycling. h) Long cycle performance at 2000 mA g^−1^ for MS@C and MS electrodes. Reproduced with permission.^[^ ^112^ ^]^ Copyright 2020, Elsevier Ltd.

In recent years, developing heterostructure has been demonstrated to be an innovative and effective strategy for promoting interfacial diffusion/reaction kinetics, this is because the built‐in electric field formed on the heterointerfaces can promote ion migration.^[^ ^105^, ^108^ ^]^ Taking MoSe_2_ as an example, it is well accepted that poor electron conductivity and sluggish K^+^ diffusion severely restrict its further application for PIBs, although it has a high theoretical specific capacity and unique layered structure. However, Yu and co‐workers^[^ ^109^ ^]^ successfully achieved an improved ions diffusion capability in MoSe_2_‐on‐NC) with the help of the built‐in electric field generated by the heterogeneous interface (**Figure** **9**a,b). They used density functional theory (DFT) calculations (Figure 9c) to further indicate that improved interface property can provide fast K‐ion migration paths with reduced diffusion barrier, and simultaneously is conducive to electron transfer (Figure 9d), leading to enhanced rate performance especially at a high current (170 mAh g^−1^ at 5000 mA g^−1^) (Figure 9e). Ex situ HRTEM images evidence that the *d*‐spacing of (002) plane in this optimized MoSe_2_ is extended to 0.92 from 0.64 nm after being fully discharged to 0.01 V, and *d* value can be recovered to 0.66 nm without obvious crystal distortion at the end of charging, highlighting a good reversibility and stability. A similar result is also verified in MoS_2_@MoO_2_ prepared by Qin and co‐workers,^[^ ^110^ ^]^ because with the finely incorporated heterojunction with elevated electron densities, the Mo—S—O and N—Mo—O channels can greatly accelerate K‐ion fast insertion/extraction, and X‐ray diffraction (XRD) analysis reveals that K ions can reversibility insert and move in (002) plane of MoS_2_, thus ensuring a good rate capability of 296 mAh g^−1^ at 500 mA g^−1^. Moreover, a MoS_2_–tungsten disulfide (WS_2_)–C microsphere containing heterogeneous interfaces is proposed,^[^ ^111^ ^]^ and the XPS and TEM test results confirm the presence of heterojunction between MoS_2_ and WS_2_. The existing heterogeneous interface not only lowers charge transfer resistance, but also promotes electronic conductivity, all of which endow an excellent rate capacity of 176 mAh g^−1^ at 5000 mA g^−1^. Cao et al.^[^ ^112^ ^]^ designed an exquisite hierarchical MoS_2_/Sb heterostructure confined into N‐doped graphene framework (MS@C), as evidenced by Figure 9f. It is found that internal MoS_2_/Sb heterostructure synergizing external nitrogen‐doped graphene aggressively accelerates electron transportation and ion diffusion, and the existence of graphene matrix can substantially stabilize SEI layer (Figure 9g), thus endowing a high rate (233.2 mAh g^−1^ at 2000 mA g^−1^) and long cycle (Figure 9h). During charge storage process, heterointerfaces can effectively restrict the coarsening of nanocrystalline and provide the buffer regions among the crystal boundaries to accommodate the extreme volume variation, leading to enhanced cycling stability. Compared to GeSe, a semiconductor‐to‐metal transition after incorporating K atoms makes GeSe/black phosphorus an enhanced conductivity,^[^ ^113^ ^]^ and it can be seen that GeSe/black phosphorus heterostructure exhibits a reduced K diffusion energy barrier on its surface. Considering the fascinating advantages of RGO (offering a high conductive channel), a self‐assembled Bi_2_S_3_ microsphere wrapped with RGO is prepared,^[^ ^108^ ^]^ where formed unique heterostructure benefits charge transfer, thus enabling a high capacity of 538 mAh g^−1^ at 200 mA g^−1^, and an impressive rate of 237 mAh g^−1^. Based on the confinement effect of dual‐carbon matrix (mainly improving conductivity, and maintaining structural integrity), Xiong and co‐workers reported small‐sized CoP nanoparticles coated with N‐doped carbon are immobilized into 1D nitrogen‐doped carbon frameworks (CoP@NC⊂NCFs),^[^ ^114^ ^]^ and they demonstrated that the interface between CoP and NC⊂NCFs gives rise to a small K‐ion diffusion energy barrier, which guarantees an extraordinary rate performance of 126 mAh g^−1^ at 10 A g^−1^. Through in situ XRD, it can be noted that there is no obvious peaks position change and new peaks present during the cycle, meaning no new phase forms. This result reveals that the potassium storage mechanism is the solid solution reaction process, that is, K ions enter the crystal lattice of CoP, but do not trigger a change in its crystal lattice, endowing a superior cyclability. With a similar purpose to Xiong and co‐workers, Sun and co‐workers^[^ ^115^ ^]^ prepared a nitrogen‐doped porous carbon confined CoP polyhedron architectures (NC@CoP/NC) to smoothly obtain a lower diffusion energy barrier on the CoP(011) surface, bringing about an improved reversible capacity of 260 mAh g^−1^ at 100 mA g^−1^. Different from Xiong and co‐workers and Sun and co‐workers, Fan and co‐workers^[^ ^116^ ^]^ synthesized a single‐carbon‐configuration‐confined ultrasmall CoP nanoparticles (CoP@NPC), and they identified that K ions can migrate freely in CoP(011) crystal plane in comparison with bulk CoP, resulting from the smallest diffusion energy barrier (0.064 eV) induced by the optimized interface between carbon matrix and CoP; thus, CoP@NPC electrode fulfills a more considerable rate capacity (229.4 mAh g^−1^ at 50 mA g^−1^) and cyclability (89.2 mAh g^−1^ over 2800 cycles at 200 mA g^−1^) than bulk CoP, but these values are slightly lower than those of CoP@NC⊂NCFs and NC@CoP/NC electrodes.

**Figure 10:**
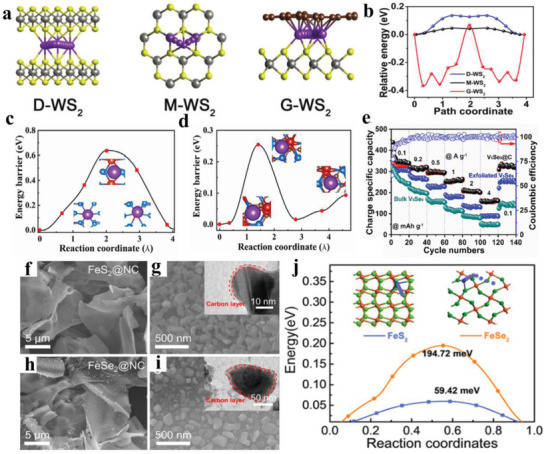
a) K^+^ migration paths and b) elative energies along the migration paths in double‐layer WS_2_ (D‐WS_2_), monolayer double‐layer WS_2_ (M‐WS_2_), and the carbon‐coated WS_2_ nanosheet (G‐WS_2_). Reproduced with permission.^[^ ^117^ ^]^ Copyright 2021, Royal Society of Chemistry. K diffusion energy barrier in c) bulk V_5_Se_8_ and d) on V_5_Se_8_ surface in V_5_Se_8_/Gr interface system. e) Rate capabilities at various current densities from 0.1 to 4000 mA g^−1^. Reproduced with permission.^[^ ^120^ ^]^ Copyright 2021, Elsevier Ltd. Field‐emission electron microscope (FESEM) images of f,g) FeS_2_@NC and h,i) FeSe_2_@NC. The insets in (g) and (i) are HRTEM images. j) Diffusion energy barrier of K on FeS_2_ and FeSe_2_ surfaces. Reproduced with permission.^[^ ^121^ ^]^ Copyright 2021, American Chemical Society.

WS_2_, as a typical 2D layered transition metal dichalcogenide (2D‐TMD),^[^ ^117^, ^118^ ^]^ exhibits high theoretical capacities and tunable interlayer spacing just like MoS_2_, MoSe_2_, etc. But its sluggish kinetics during K‐ion migration process usually leads to inferior rate performance. In this regard, Zhou and co‐workers^[^ ^117^ ^]^ significantly extended rate capacity of WS_2_ to 168 mAh g^−1^ at 10 A g^−1^ by designing carbon‐coated WS_2_ nanosheets supported on carbon nanofibers (C–WS_2_@CNFs). They ascribed this result to the improved K‐ion transfer ability at the interface of the C–WS_2_@CNF nanosheets, as evidenced by DFT calculations that G‐WS_2_ has the lowest diffusion barrier (**Figure** **10**a,b). The presence of carbon configurations can well accommodate structure strain induced by K‐ion insertion, thus crystal or phase structure of WS_2_ is considerably preserved, and therefore can stably store K via reversible conversion reaction. As an analog of WS_2_, WSe_2_ also exhibits a relatively fascinating promising for PIBs, but it still has an inferior solid/solid interface ability for K ions’ freely diffusion.^[^ ^119^ ^]^ Note that the incorporation of nitrogen‐doped carbon coating layer can effectively improve electronic conductivity of WSe_2_ and strengthen the structural stability,^[^ ^119^ ^]^ and DFT computation reveals that the introduced carbon layer effectively optimizes the interface between it and WSe_2_, noticeably making the K‐diffusion barriers lower than that of bulk WSe_2_ without carbon coating. Resultantly, the rate performance at 1000 mA g^−1^ is greatly increased from 18 mAh g^−1^ for bulk WSe_2_ to 231 mAh g^−1^ for WSe_2_ with desirable interface ability. Another layered 2D‐TMD (V_5_Se_8_@C nanosheets) is also developed to improve its unsatisfactory charge transfer ability,^[^ ^120^ ^]^ and it is found that V_5_Se_8_/graphene interface system has a smaller energy barrier (0.25 eV) compared to bulk V_5_Se_8_ (0.64 eV) (Figure 10c,d). This result means that V_5_Se_8_@C can realize a fast K‐ion diffusion in the interface as expected, and the calculated diffusion coefficient (1.08 × 10^−7^ cm^2^ s^−1^) is much higher than that of bulk V_5_Se_8_ (8.40 × 10^−14^ cm^2^ s^−1^). Such a superior K‐ion diffusion kinetics is highly related to heterogeneous structure induced by the introduction of carbon sheets into V_5_Se_8_, which favors a high‐rate capability with a capacity of 162 mAh g^−1^ at 4000 mA g^−1^ (Figure 10e). Xi and co‐workers^[^ ^121^ ^]^ reported nitrogen‐doped carbon‐coated FeS_2_ (FeS_2_@NC) and FeSe_2_ (FeSe_2_@NC) (Figure 10f–i), and they discovered that the introduction of carbon species decreases the K‐diffusion energy barrier for both FeS_2_ and FeSe_2_ (Figure 10j). Accordingly, the improved interface between FeS_2_, FeSe_2_, and carbon can markedly facilitate ion diffusion, thus endowing enhanced rate performance (154.7 mAh g^−1^ for FeS_2_@NC vs 133.2 mAh g^−1^ for FeSe_2_@NC). Simultaneously, they also pointed out that the slightly lower capacity of FeSe_2_@NC is because the Se atom with a larger radius blocks ion transfer paths to some extent, as observed in Figure 10j. As for charge storage process, in situ XRD and ex situ TEM analysis confirm the formation of several intermediate phases (K*_x_* FeS_2_) that have high conductivity and large interlayer distance, which promote reversible potassium insertion and facilitate charge transfer. Bao and co‐workers^[^ ^122^ ^]^ combined Schottky junction and multi‐heterostructure to synergistically enhance rate performance and cycling stability of SnS@C@MoS_2_@NC anode. In this study, they demonstrated that the existence of Schottky junction induced by phase boundaries can boost intrinsic electronic conductivity, and the multi‐heterostructure results from the introduction of double‐carbon layer exhibits low diffusion barriers, which enables fast charge transfer and therefore favors an outstanding rate performance (305 mAh g^−1^ at 1000 mA g^−1^).

From the above discussion, we can well know that K‐ion diffusion in the solid‐state electrode is very sluggish, due to a high charge transport impedance, thus usually causing inferior rate performance, especially at a high current density. Fortunately, it is found that the design of heterostructure can effectively lower K‐diffusion energy barrier by optimizing interface properties of active materials, and this conclusion is often verified by DFT calculations. As a result, an excellent rate capacity is easily obtained in electrode with improved interface ability, which is noticeably superior to bulk electrode without heterogeneous interface.

### 3.2 Expanded Interlayer Spacing

**Figure 11:**
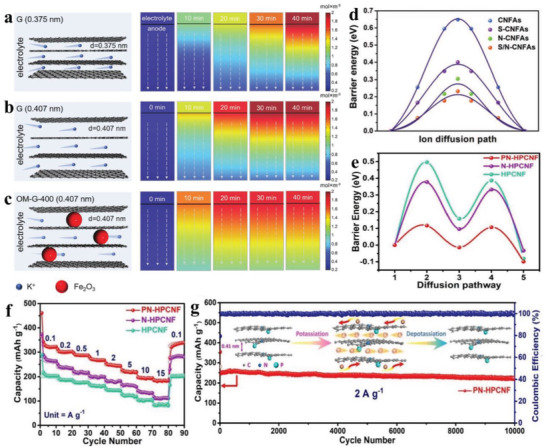
COMSOL Multiphysics simulations of K‐ion transport kinetics in a) G (0.375 nm), b) G (0.407 nm), and c) OM‐G‐400 (0.407 nm) anodes at different discharging times. Reproduced with permission.^[^ ^124^ ^]^ Copyright 2021, Wiley‐VCH. d) K‐ion diffusion barriers of CNFAs, S–CNFAs, N–CNFAs, and S/N–CNFAs. Reproduced with permission.^[^ ^125^ ^]^ Copyright 2019, Wiley‐VCH. e) K‐ion diffusion barriers of hierarchical porous carbon nanofibers (HPCNF), N‐doped hierarchical porous carbon nanofibers (N–HPCNF), and P/N co‐doped hierarchical porous carbon nanofibers (PN–HPCNF). f) Rate capabilities and g) cycling performance of HPCNF, N–HPCNF, and PN–HPCNF. Reproduced with permission.^[^ ^126^ ^]^ Copyright 2021, Royal Society of Chemistry.

When K ions insert into the electrode, the ion migration rate is usually affected by interlayer or crystal plane spacing. If K ions are squeezed into a confined interlayer spacing, a huge transport barrier and slow electrochemical reaction kinetics will be inevitably encountered. For example, graphite has been demonstrated as an anode for PIBs by forming potassium‐intercalation compounds with a theoretical capacity of 279 mAh g^−1^,^[^ ^123^ ^]^ but its narrow interspace of 0.335 nm cannot provide sufficient space to accelerate K‐ion diffusion, leading to poor rate performance, especially rate capacity at 200 mA g^−1^ is less than 50 mAh g^−1^.^[^ ^123^ ^]^ Therefore, increasing interface distance between layers to reduce K‐ion migration energy barrier is regarded as a good method, and has been widely reported. A research on the influence of different interlayer interface spaces on K‐ion transport kinetics was evaluated by Xu and co‐workers,^[^ ^124^ ^]^ and they well established the correlation between K‐ion diffusion kinetics and interplanar space through COMSOL Multiphysics simulations, that is, K‐ion migration rate increases with increasing interplanar space from 0.375 to 0.407 nm (**Figure** **11**a,b). They also pointed out that K‐ion migration rate can be further increased by modifying interface between layers with Fe_2_O_3_ nanoparticles (Figure 11c), this is mainly because the lyophilic effect of Fe_2_O_3_, thus enabling electrochemical performance in terms of rate (306.6 mAh g^−1^ at 10 A g^−1^) and cycle (384.8 mAh g^−1^ after 2000 cycles at 1000 mA g^−1^). Some previous reports have proved that heteroatom doping not only creates extra active sites for K‐ion adsorption storage, but expands distance between layers, the latter are expected to reduce K^+^ diffusion energy barrier for intercalation storage.^[^ ^125^, ^126^ ^]^ Yang and co‐workers^[^ ^125^ ^]^ comparatively demonstrated that S/N codoping is beneficial to synergistically improve K‐ion transport kinetics in S/N codoped carbon nanofiber aerogels (S/N‐CNFAs), and they well identified this conclusion through the DFT calculations, in which they discovered that S/N–CNFAs with an interlayer spacing of up to 0.408 nm has the smallest diffusion energy barrier (0.23 eV) compared to CNFAs (0.64 eV), S–CNFAs (0.38 eV), and N–CNFAs (0.30 eV) (Figure 11d). Benefiting from the excellent improved transfer dynamic induced by expanded interlayer spacing, S/N–CNFAs deliver the best rate capacity (112 mAh g^−1^ at 5000 mA g^−1^) and cycling stability. A similar phenomenon is also observed by Wen and co‐workers,^[^ ^126^ ^]^ who found that interlayer space of CNFs can be expanded by doping P and N heteroatoms, and when P/N heteroatoms are simultaneously embedded into CNF, an interlayer spacing of as high as 0.408 nm is obtained. Therefore, P/N‐codoped CNF delivers the lowest diffusion energy barrier (0.12 eV) in comparison with other two samples (Figure 11e), which enables outstanding rate performance of 184 mAh g^−1^ at 15 A g^−1^ (Figure 11f), and ultralong lifespan over 10 000 cycles at 2000 mA g^−1^ (Figure 11g). Ci and co‐workers^[^ ^12^ ^]^ illustrated that K‐transport energy barrier gradually decreases with increasing interlayer distances, and they theoretically found that the minimum energy barrier (≈0 eV) is achieved when interlayer spacing is 0.52 nm. With the superiority of interlayer engineering in improving K‐ion diffusion kinetics demonstrated, the prepared KC electrode, that has an expanded interlayer (0.417 nm), delivers a prominent rate of 158.6 mAh g^−1^ at 10 A g^−1^. As aforementioned, MoS_2_ has a tunable interlayer spacing, which means that realizing a fast K‐diffusion rate is feasible by modulating interlayer spacing. Based on this purpose, Li and co‐workers^[^ ^127^ ^]^ designed a composite of MoS_2_ and N/O‐doped carbon to successfully expand interlayer distance of MoS_2_ to 0.92 nm. Based on the DFT calculations, they signified that the expansion of interlayer spacing can reduce the diffusion energy barriers of K^+^ in the interlayer of electrode material, thus ensuring 247 mAh g^−1^ at 1000 mA g^−1^.

The beneficial effects of enlarged interlayer spacing are mainly to reduce K‐migration resistance in solid‐state diffusion. Heteroatom doping is a meaningful approach to increase interlayer spacing of carbon materials, and the as‐obtained products have an obvious improved K‐ion diffusion rate in comparison with their counterpart undoped samples, which usually is evidenced by the DFT calculation and GITT test results. As for MoS_2_, it has been demonstrated that the expansion of interlayer spacing can allow K ions to freely transport or diffuse, which greatly avoids sluggish reaction dynamic, leading to increased rate performance especially at a high current density.

### 3.3 Other Strategies

**Figure 12:**
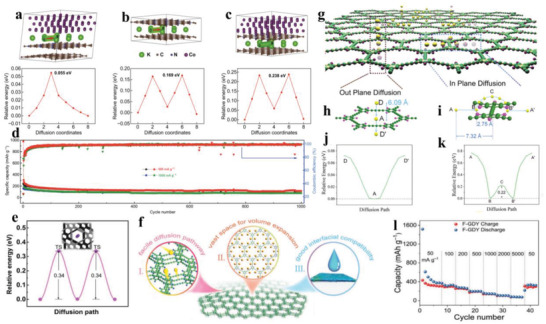
The corresponding barrier energy for K ions diffusing a) at the interface of cobalt nanoparticles and NC, b) between the carbon layers of NC, and c) between the carbon layers of Co–NC. d) Long‐term cycling performance and the corresponding CE of Co–NC anode at 500 and 1000 mA g^−1^. Reproduced with permission.^[^ ^130^ ^]^ Copyright 2021, Springer. e) Diffusion path of K ion within graphdiyne cavity and the corresponding energy profile. Reproduced with permission.^[^ ^132^ ^]^ Copyright 2020, Wiley‐VCH. f) Structure and morphology investigation of the dense F‐GDY film. g) The schematic K atom diffusion pathway in F‐GDY single layer. h,i) The out plane diffusion pathway and the corresponding energy of K atom in F‐GDY layer. j,k) The plane diffusion pathway and the corresponding energy of K atom in F‐GDY layer. l) The rate of performance of F‐GDY at different current densities. Reproduced with permission.^[^ ^128^ ^]^ Copyright 2020, Wiley‐VCH.

Designing heterostructure and expanding interlayer spacing are meaningful to lower the K‐diffusion energy barrier by improving interface properties, and therefore largely promote rate performance. However, some other research strategies on controlling interface properties are also reported in PIBs. Among them, introducing second phase atom into host materials, and developing a new electrode material based on the experimental tests and first principle calculations are commonly investigated.^[^ ^128^, ^129^, ^130^ ^]^ Actually, the former can form chemical bonding between second phase atom and host materials, while the latter is beneficial to obtain optimized layer structure, all of which can reduce diffusion barriers and therefore accelerate interfacial diffusion of K ions. Zhou and co‐workers^[^ ^130^ ^]^ smoothly incorporated highly dispersed Co nanoparticles into nitrogen‐doped graphitized carbon (Co–NC) using Prussian blue analog as raw materials. They claimed that formed Co—C bonds contribute to enhancing interface performance between Co and N‐doped graphitized carbon, and based on the DFT calculation results (**Figure** **12**a–c), they further discovered that this optimized interface significantly decreases the diffusion energy barrier when K ions pass through, thus enabling an enhanced rate capability (175.4 mAh g^−1^ at 2000 mA g^−1^). Besides, the presence of Co—C bonds is also proved to promote structure stability, as evidenced by the cycling performance in Figure 12d. Combining with cyclic voltammetry (CV), ex situ XRD patterns, and mapping, they demonstrated that Co nanoparticles are not alloyed with K^+^ during the charge and discharge processes, emphasizing that Co nanoparticles only modify interface without other effects. Different from the enlarged interlayer spacing induced by P‐doping, Qian and co‐workers^[^ ^131^ ^]^ found that the introduction of P into carbon matrix can accelerate ions fast diffusion via formed P—O bonds. Through DFT calculations, they illustrated that the diffusion barriers of ions along P—O‐functionalized paths are smaller than that of P—C paths. Therefore, the resulting carbon electrode with the optimal P—O/P—C ratio displays an exceptional rate capacity of 168 mAh g^−1^ at 5000 mA g^−1^, and an extraordinary long cycle performance at 1000 mA g^−1^ over 3000 cycles. Recently, graphdiyne (GDY) composed of sp and sp^2^ hybrid carbon atoms, as a new 2D carbon allotrope, has been demonstrated to be an excellent electrode in LIBs and SIBs, owing to its unique advantages of good conductivity and facilitated ion diffusion pathway.^[^ ^128^, ^132^ ^]^ When this GDY is applied in PIBs, Sun and co‐workers^[^ ^132^ ^]^ found that K atom prefers to stay at the cavity center of GDY, due to a quite low K‐ion diffusion barrier (Figure 12e). They also revealed that the presence of versatile pore structure facilitates the K‐ion transport through the interlayer spacing, which benefits a good rate capability. Also, Wei and co‐workers^[^ ^129^ ^]^ indicated that large hexagonal C‐rings make K^+^ quickly across the *α*‐graphdiyne layer by providing a smaller diffusion barrier (0.353 eV). Huang and co‐workers^[^ ^128^ ^]^ fabricated fluoride graphdiyne (F‐GDY) (Figure 12f), and unveiled that K atoms can diffuse easily both in plane and out of plane in F‐GDY layers, as evidenced by lower barriers (0.22 eV, in plane; 0.07 eV, out plane) (Figure 12g–k). Notably, they attributed the improved K‐diffusion ability to the good interfacial compatibility formed after introducing F atom into GDY, thus endowing 75 mAh g^−1^ at 5000 mA g^−1^ (Figure 12l).

Introducing second phase atom is of very significance for improving K‐ion diffusion capability in solid‐state electrode, resulting in improved rate, but the related researches are still poor. As an ideal second phase doping atom, it is required to only improve diffusion/reaction kinetics without other adverse effect, thus more characterization techniques, such as ex situ XPS, XRD, EDS, and mapping, need to be performed to explore whether the second phase atoms will react with active potassium ions. DFT calculation is quite important and indispensable for developing a new electrode material; through it, we can intuitively observe the K‐diffusion paths and the corresponding energy barrier, which is conducive to further optimize electrode structure with the aim of achieving superior interface properties.

## 4 Summary and Prospects

Attracted by the unique advantages of low cost and high operating potential, PIBs have obtained enormous attention and are expected to store intermittent electricity for smart grid regulation. During the electrochemical reaction process, K^+^ first passes through the SEI layer, and then arrives at the surface of active materials, and finally stores in crystal structure via solid‐state diffusion in electrode. Through the overall charge storage process, we can clearly see that SEI layer interface (known as solid–liquid interface) and crystal interface (termed as solid–solid interface) are quite important procedures for K^+^ transport. To be specific, a robust and uniform SEI film is highly desirable, which is because it can insulate electrons, ensure K^+^ transfer, suppress electrolyte depletion, and maintain electrode structural integrity. In terms of crystal interface, it significantly affects the K‐migration rate between crystals, and hence determines final rate performance. Generally, the optimization of electrolyte systems (such as potassium salts, solvent, concentration, and additives) and binders (CMCNa and PAANa) can contribute to forming a dense and stable SEI layer, leading to improved ion transfer capability and structure stability. It has been demonstrated that designing the heterogeneous interface and expanding the interlayer spacing can effectively reduce diffusion energy barrier, giving rise to an increased K‐migration rate. Accordingly, it is very necessary to in‐depth understand solid–liquid interface and solid–solid interface. In this work, we analyze the effect mechanism of different improvement strategies on constructing stable SEI layer and enhancing diffusion ability of K ions within electrode, which contributes to improving electrochemical performance (**Tables** **3** and **4**). Although some progress has been made, the further development for interface properties in PIBs still faces several challenges.

1) Potassium salts and solvents have a substantial effect on the formation of SEI, and the obtained SEI has different properties. For inserted type graphite anodes, ether solvents (DEGDME and DME) tend to form a negligible SEI film, and this SEI can be well maintained due to a small volume expansion induced by [K–solvent]^+^ complexes’ cointercalation. While the decomposition of EC/DEC‐ or EC/PC‐based electrolyte causes the overgrowth of SEI, which is not only present in graphite, but can also be observed in conversion and alloying type anodes. Besides, the use of KFSI‐based electrolyte is more conducive to form an elastic and continuous SEI layer composed of organic species, which can suppress the exposure of new active particle surfaces, especially for conversion and alloying type anodes. So, the evolution from KPF_6_ to KFSI is a growing trend in constructing a stable SEI on anode, but more attention is required to be paid to explore the mechanism of decomposition and film formation of KFSI.
2) As aforementioned, both potassium salts and solvents have a substantial effect on the properties of SEI, therefore, how to evaluate the proportion of these two components in the formation of SEI is important. According to LUMO,^[^ ^31^ ^]^ it can be clearly found that the order of LUMO level is KFSI (−2.062 eV) < KPF_6_ (−1.988 eV) < EC (−0.433 eV) < EMC (≈0 eV) < DME (1.177 eV), implying the lowest reduction stability of KFSI. Based on this, regardless of the solvent used, the obtained SEI is mainly derived from the preferential decomposition of KFSI, hence KFSI salt has a more substantial effect on SEI compared to solvents. For KPF_6_‐based electrolyte, the used solvents (e.g., EC, DEC, and DME) always tend to decompose to form SEI films with organic and inorganic species, indicating that solvents instead of KPF_6_ salt are more important in determining SEI formation.
3) Because anodes for PIBs generally exhibit a large volume change and SEI instability, PIBs will exhibit an obvious improved interfacial performance if suitable functional binders that solve these limitations are selected. Compared to PVDF, there are many hydroxyl groups present in CMCNa, which makes it an enhanced adhesiveness, while PAANa has a high surface coatability and mechanical strength. All these are favorable for forming stable SEI, but the related research is still lacking and needs to be paid more attention. The construction of hollow structure and core–shell structure can alleviate volume change, and thus protect SEI from repeated forming, leading to enhanced interface properties; however, the presence of numerous voids space is unfavorable for exerting the merits of volume energy density in a K full cell. Therefore, keeping a balance between excellent interface properties and volume energy density is essential for further application of PIBs.
4) Given that excellent electrical conductivity and ion migration abilities enabled by built‐in electric field, designing heterostructure is a good choice to boost crystal interfacial properties. Heterostructure materials are usually synthesized by vapor–solid reaction method, hydrothermal method, sol–gel method, etc. Nevertheless, interfacial transmission mechanism is only revealed by DFT calculation, which is insufficient to comprehensively demonstrate the reasons that enhance electrochemical performance, thus using some in situ or ex situ characterization technologies is necessary. Particularly, after carbon modification, the interface properties of CoP and V_5_Se_8_ are also greatly enhanced, but once the economic cost is taken into consideration, making scale production difficult. Notably, heteroatom doping is a meaningful method to expand interlayer spacing, and as a result, the K‐diffusion energy is significantly lowered, thus endowing an improved K‐ion transport rate within active materials. As for the introduction of second phase atom, the synthesis strategies are similar to heteroatom doping to some extent, while it usually reacts with host materials to form chemical bonds, such as Co—C and P—O bonds, which can well lower interfacial diffusion energy barrier and improve structure stability. Therefore, exploring suitable second phase atoms and introducing them into host active materials could be a good choice to optimize interfacial behaviors, including ion diffusion, Coulombic efficiency, and interfacial impedance.

**Table 3:** Summary on electrochemical performance of some reported electrode materials

Strategies	Materials	Electrolyte	Potential range [V]	Rate [mAh g^−1^/A g^−1^]	Cycle [mAh g^−1^/A g^−1^/cycles]	Ref.
Forming stable SEI	NGFs	0.6 m KFSI in 1:1 v/v EC/DEC	0.01–1.5	115/0.16	–	^[^ ^42^ ^]^
	NiCo_2.5_S_4_	1 m KFSI in 1:1 v/v EC/PC	0.01–3.0	402/2	495/0.2/1900	^[^ ^43^ ^]^
	Graphite oxide	0.8 m KPF_6_ in 1:1 v/v EC/DEC	0.01–3.0	87.1/1	63.6/1/1000	^[^ ^51^ ^]^
	Graphite	0.8 m KPF_6_ in DME	0.01–2.0	87/2.8	94/2.8/3800	^[^ ^56^ ^]^
	HC	1 m KFSI in 1:1 v/v EC/DEC	0.01–3.0	–	103/0.1/550	^[^ ^41^ ^]^
	FeP@FGCS	1 m KFSI in DME	0.01–3.0	221/2	183/3/1000	^[^ ^59^ ^]^
	O‐doped carbon	3 m KFSI in DME	0.01–2.5	145/2	183/0.5/3000	^[^ ^63^ ^]^
	Graphite	2.76 mol kg^−1^ KFSI/DME–HFE	0.01–2.0	–	200/0.025/300	^[^ ^70^ ^]^
	Graphite	1.0 m KFSI in TMP	0.01–2.0	228/0.672	272/0.1/100	^[^ ^80^ ^]^
	Fe_3_O_4_/3DNPGF	1 m KFSI in DME	0.01–3.0	97/2	154.6/1/500	^[^ ^93^ ^]^
	HCS‐600	1 m KPF_6_ in 1:1 v/v EC/P	0.01–3.0	130/1	111/1/3000	^[^ ^82^ ^]^
	UCF@CNs@BiNs	3 m KFSI in DME	0.01–3.0	140/1	372/0.1/600	^[^ ^98^ ^]^
	MoS_2_/N‐doped‐C	0.8 m KPF_6_ in 1:1 v/v EC/DEC	0.01–2.5	131/2	151/1/500	^[^ ^99^ ^]^
	Bi_2_Se@NC@RGO	5 m KFSI in 1:1 v/v EC/DMC	0.01–3.0	101.6/5	113.5/0.5/1000	^[^ ^100^ ^]^
	Co_3_Se_4_/GO	1 m KFSI in DME	0.01–3.0	256.7/2	301.8/1/500	^[^ ^103^ ^]^
	Fe_3_C@PGC–NGF	1 m KPF_6_ in 1:1 v/v EC/DEC	0.01–2.5	195/1	155/1/1000	^[^ ^102^ ^]^
	Cu_2_S@NC	5 m KFSI in DME	0.01–2.5	257/6	317/1/1200	^[^ ^104^ ^]^
	Carbon fiber	1 m KPF_6_ in 1:1 v/v EC/DMC	0.01–2.5	177.3/2.5	171.5/1.5/500	^[^ ^23^ ^]^
Heterointerface	MoSe_2_‐on‐NC	0.7 m KPF_6_ in 1:1 v/v EC/DEC	0.01–3.0	170/5	247/1/2800	^[^ ^109^ ^]^
	MoS_2_@MoO_2_	0.5 m KPF_6_ in 1:1 v/v EC/PC	0.01–2.5	295/0.5	–	^[^ ^110^ ^]^
	MoS_2_–WS_2_	1 m KFSI in DME	0.01–3.0	176/5	350/0.2/100	^[^ ^111^ ^]^
	MS@C	1 m KFSI in DME	0.01–3.0	233.2/2	170.1/2/1000	^[^ ^112^ ^]^
	Bi_2_S_3_@RGO	1 m KPF_6_ in DME	0.01–2.5	–	237/2/300	^[^ ^108^ ^]^
	CoP@NC⊂NCFs	1 m KPF_6_ in 1:1 v/v EC/DEC	0.01–2.5	126/10	205.7/100/1200	^[^ ^114^ ^]^
	NC@CoP/NC	0.8 m KPF_6_ in 1:1 v/v EC/DEC	0.01–2.5	–	260/0.1/100	^[^ ^115^ ^]^
	CoP@NPC	0.8 m KPF_6_ in 1:1 v/v EC/DEC	0.01–3.0	229.4/0.05	89.2/0.2/2800	^[^ ^116^ ^]^
	C–WS_2_@CNFs	1 m KFSI in 1:1 v/v EC/DMC	0.01–3.0	168/10	168/2/300	^[^ ^117^ ^]^
	V_5_Se_8_@C	1 m KFSI in 1:1 v/v EC/DMC	0.01–2.5	162/4	145/4/800	^[^ ^120^ ^]^
	FeS_2_@NC	1 m KFSI in 1:1 v/v EC/DEC	0.01–2.5	154.7/10	73.6/2/5000	^[^ ^121^ ^]^
Expanding interspacing	Graphene	1 m KFSI in 1:1 v/v EC/DEC	0.01–3.0	306.6/10	384.8/1/2000	^[^ ^124^ ^]^
	S/N–CNFAs	1 m KPF_6_ in 1:1 v/v EC/DMC	0.01–2.5	122/5	168/2/1000	^[^ ^125^ ^]^
	P/N–CNF	1 m KFSI in DME	0.01–3.0	184/15	226/2/10 000	^[^ ^126^ ^]^
	KC	0.8 m KPF_6_ in 1:1 v/v EC/DEC	0.01–3.0	156/10	151.2/5/2000	^[^ ^12^ ^]^
Second phase atoms	Co–NC	0.8 m KPF_6_ in 1:1 v/v EC/DEC	0.01–3.0	175.4/2	78.5/1/1000	^[^ ^130^ ^]^
	P—C	0.8 m KPF_6_ in 1:1 v/v EC/DEC	0.01–3.0	168/5	218/1/3000	^[^ ^131^ ^]^
Exploring new materials	F‐GDY	3 m KFSI in DEC with 5% FEC	0.01–3.0	75/5	120/1/1800	^[^ ^128^ ^]^

**Table 4:** Summary on mass loading, capacity retention, and inner resistance of mentioned electrode materials

Materials	Mass loading [mg cm^−1^]	Capacity retention [%]	Interface resistance [Ω]	Ref.
NGFs	1.9	81.0	26.6–55.9	^[^ ^42^ ^]^
Graphite oxide	1.0	–	48.8	^[^ ^51^ ^]^
Graphite	2.0	87.4	10.0	^[^ ^56^ ^]^
FeP@FGCS	1.0	88.3	118.7	^[^ ^59^ ^]^
O‐doped carbon	4.5	91.5	–	^[^ ^63^ ^]^
Graphite	1.0	89.4	–	^[^ ^70^ ^]^
Fe_3_O_4_/3DNPGF	0.8–1.0	98.5	–	^[^ ^93^ ^]^
MoS_2_/N‐doped‐C	1.0	74.0	–	^[^ ^99^ ^]^
Bi_2_Se@NC@RGO	0.6–0.8	76.2	–	^[^ ^100^ ^]^
Cu_2_S@NC	1.0	83.2	580.0	^[^ ^104^ ^]^
MoS_2_@MoO_2_	–	91.0	–	^[^ ^110^ ^]^
MoS_2_–WS_2_	1.4	–	–	^[^ ^111^ ^]^
MS@C	0.8	94.6	8.448	^[^ ^112^ ^]^
Bi_2_S_3_@RGO	1.0–1.2	–	5.84	^[^ ^108^ ^]^
CoP@NC⊂NCFs	1.0	86.79	479.9	^[^ ^114^ ^]^
NC@CoP/NC	1.33	70.0	–	^[^ ^115^ ^]^
CoP@NPC	0.8–1.0	–	–	^[^ ^116^ ^]^
C–WS_2_@CNFs	1.0	91.0	917.0	^[^ ^117^ ^]^
V_5_Se_8_@C	1.2	82.9	–	^[^ ^120^ ^]^
FeS_2_@NC	–	–	–	^[^ ^121^ ^]^
Graphene	1.5	96.3	–	^[^ ^124^ ^]^
S/N–CNFAs	1.4	–	–	^[^ ^125^ ^]^
P/N–CNF	0.8–1.0	92.1	–	^[^ ^126^ ^]^
KC	1.0–1.2	83.2	–	^[^ ^12^ ^]^
P—C	2.0–6.0	78.9	–	^[^ ^131^ ^]^
F‐GDY	0.25	–	–	^[^ ^128^ ^]^

Overall, PIBs are becoming a very competitive candidate for next‐generation energy storage devices. Exploring interfacial transmission mechanism and optimizing interfacial structure have been demonstrated to improve interfacial transfer ability, favoring excellent rate performance. Although some pioneering works have been performed, we intensively believe that significant research progress is still needed for real commercial application.

## Conflict of Interest

The authors declare no conflict of interest.
